# Immunocytochemical Analysis of Crocin against Oxidative Stress in Trigeminal Sensory Neurons Innervating the Cornea

**DOI:** 10.3390/molecules29020456

**Published:** 2024-01-17

**Authors:** Cristina Sánchez-Fernández, Susana Del Olmo-Aguado, Enol Artime, Alberto Barros, Luis Fernández-Vega Cueto, Jesús Merayo-Lloves, Ignacio Alcalde

**Affiliations:** 1 Instituto Universitario Fernández-Vega, Fundación de Investigación Oftalmológica, Universidad de Oviedo, 33012 Oviedo, Spain; cristina.sanchez@fio.as (C.S.-F.); solmo@fio.as (S.D.O.-A.); enol.artime@fio.as (E.A.); alberto.barros@fernandez-vega.com (A.B.); lfvc@fernandez-vega.com (L.F.-V.C.); merayo@fio.as (J.M.-L.); 2 Instituto de Investigación Sanitaria del Principado de Asturias (ISPA), 33011 Oviedo, Spain

**Keywords:** crocin, saffron, corneal innervation, neuroprotection

## Abstract

Corneal diseases are a major cause of vision loss, often associated with aging, trauma and disease. Damage to corneal sensory innervation leads to discomfort and pain. Environmental stressors, such as short-wavelength light, can induce oxidative stress that alters mitochondrial function and affects cell and tissue homeostasis, including corneal innervation. Cellular antioxidant mechanisms may attenuate oxidative stress. This study investigates crocin, a derivative of saffron, as a potential antioxidant therapy. In vitro rat trigeminal sensory ganglion neurons were exposed to both sodium azide and blue light overexposure as a model of oxidative damage. Crocin was used as a neuroprotective agent. Mitochondrial and cytoskeletal markers were studied by immunofluorescence analysis to determine oxidative damage and neuroprotection. In vivo corneal innervation degeneration was evaluated in cornea whole mount preparations using Sholl analyses. Blue light exposure induces oxidative stress that affects trigeminal neuron mitochondria and alters sensory axon dynamics in vitro, and it also affects corneal sensory innervation in an in vivo model. Our results show that crocin was effective in preserving mitochondrial function and protecting corneal sensory neurons from oxidative stress. Crocin appears to be a promising candidate for the neuroprotection of corneal innervation.

## 1. Introduction

Corneal diseases represent the most prevalent causes of ophthalmology consultations worldwide and are the fourth most common cause of vision loss [1].

Despite its apparently simple anatomy, the cornea is one of the most densely innervated tissues in the human body, mainly comprising sensory and autonomic nerve fibers [2,3]. In addition to the importance of their sensory functions, corneal nerves help maintain the functional integrity of the ocular surface by regulating tear production, releasing trophic substances and promoting tear and epithelial homeostasis [4,5,6].

The cornea is also a barrier that protects the internal structures of the eye from infections, trauma and exogenous environmental insults, including UV light (mainly UVB), ionizing radiation and pollution toxins. Prolonged exposure to these exogenous insults contributes to oxidative damage to most cells and tissues [7]. An imbalance between reactive oxygen species (ROS) generation and the capacity of antioxidant ROS scavenging systems results in oxidative stress associated with cell damage, such as lipid peroxidation of membranes, oxidative changes in proteins, and oxidative damage to DNA [8]. Oxidative stress and DNA damage have been associated with the degeneration of the primary sensory neurons innervating the cornea related to the aging senescence process [5,9], suggesting that both the cornea and corneal innervation would be highly susceptible to damage from ROS [8].

It is well known that light in the retina (approximately 400–780 nm) is absorbed by enzyme complexes of the electron transport system of mitochondria [10]. Apart from UV radiation, short-wavelength light (SWL), defined as being between 400 and 450 nm in the blue/violet part of the spectrum [11,12], negatively affects mitochondrial functions [10]. Progressive accumulation of mutations in mitochondrial DNA (mtDNA) reduces ATP production and increases ROS generation, thus driving oxidative stress, inflammation and cell loss [10,13]. Mitochondria, as the main sources of energy production, play a major role in causing inflammation and oxidative stress [10,14].

In healthy corneas, a number of antioxidant protective mechanisms are present to minimize and reduce the negative effects of UV [15]. Aldehyde dehydrogenase (ALDH3) directly absorbs UV and removes cytotoxic aldehydes. Furthermore, the cornea reveals antioxidant enzymes, such as superoxide dismutase (SOD), catalase (CAT) and glutathione peroxidase (GPx) scavenging ROS [8,15]. When its protective mechanisms are overwhelmed due to the excessive amount of ROS, the cornea may be disturbed by toxic oxygen products [15].

SWL from 410 to 480 nm (blue light) has been demonstrated to induce cell death in cultured transformed cells (RGC-5 cells) involving the generation of ROS and the activation of poly-(ADP-ribose) polymerase (PARP) and apoptosis-inducing factor (AIF) [10,16,17], and it also induces inflammatory and oxidative stress markers in primary trigeminal neurons and glia in culture [18]. In addition to superoxide and hydrogen peroxide generation [18], activation of hemoxygenase-1 (HO-1), a stress-response antioxidant enzyme, has been extensively described in relation to blue light photooxidation [17]. HO-1 is an early indicator of oxidative stress but also acts as a regulatory mechanism for oxidative damage. Damage to corneal nerve endings, whether caused by natural aging, mechanical trauma or by ocular and systemic diseases, can lead to discomfort, pain and long-term damage to the integrity of the ocular surface [19,20,21].

Recently, an article by Gao et al. [22] also demonstrated that exposure to blue light caused increased hyperalgesia because of the damage of the sensory nerves in a mouse cornea. These authors also observed a reduction in tear secretion in mice exposed to blue light, suggesting the generation of a dry eye disease (DED) scenario. Patients suffering from DED showed a marked increase in inflammatory activity in the tear film as well as lipid peroxide and myeloperoxidase activity, which are indicators of oxidative tissue damage [23]. Free radicals and inflammation may be involved in the pathogenesis or in the self-propagation of the DED [8,15].

Antioxidant treatments have been used to treat corneal diseases, mainly related to inflammatory conditions, such as DED [23] and corneal ulceration [24], indicating that topical antioxidant therapy was effective in reducing the inflammatory corneal reaction. One of the most interesting compounds showing antioxidant properties is saffron from *Crocus sativus* [25,26]. Due to its efficacy in neurodegenerative diseases [27,28], saffron has been used in the treatment of retinal diseases, such as age related macular degeneration, diabetic retinopathy and glaucoma [29,30,31].There is a growing interest in the isolated components of saffron because of their antioxidant and anti-inflammatory functions on human health due to their free radical scavenging activity [25]. The most bioactive compounds of saffron are safranal, crocetin, picrocrocin and crocin [25]. Crocin has been suggested to have antioxidant, anti-inflammatory, and even anti-cancer properties [25,32]. Crocin modulates oxidative stress mostly by scavenging free radicals and stimulating the expression of antioxidant enzymes including SOD, GPx, glutathione S-transferase (GST) and CAT, as well as a reduction in malonyldialdehyde (MDA) and lipid peroxidation [32].

In this work, we present an in vitro and in vivo study of the oxidative effects of blue light overexposure on the sensory neurons innervating the cornea, and the evaluation of crocin as an antioxidant therapy.

## 2. Results

To evaluate if blue light has an oxidative damage effect on the sensory innervation of the ocular surface, we used neuronal primary cultures from rat trigeminal ganglia exposed to a source of 18 W/m^2^ LED light emitting at 470 nm (blue light). As a control for the damage condition, we used 5 mM sodium azide (NaN_3_), as it is a known oxidant affecting the complex IV of the mitochondrial electron transport chain.

To evaluate the antioxidant and neuroprotective efficacy of crocin, we pretreated the cultures with 0.05 mM crocin for 24 h before NaN_3_ addition and blue light exposure.

We further evaluate the oxidative effect of blue light and neuroprotective efficacy of crocin on the sensory neurons innervating the cornea in an in vivo procedure. The neuroprotectant (0.1 M crocin) was applied topically for 3 days before the beginning of blue light exposure and for the duration of experiment.

### 2.1. Blue Light Induces Oxidative Stress and Mitochondrial Dysfunction in Rat Trigeminal Ganglion Neurons in Culture

**Figure 1:**
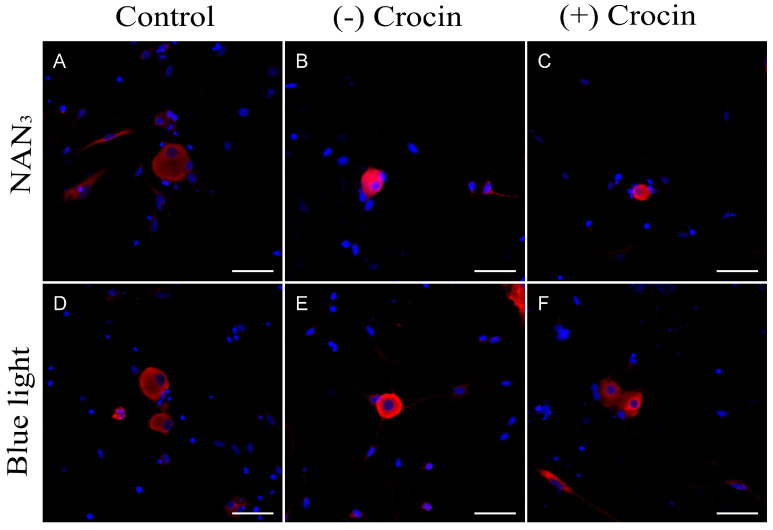
HO−1 protein detection in undamaged neurons from primary cultures of trigeminal ganglia (**A**,**D**) and after exposure to NaN_3_ (**B**) and blue light (**E**). (**C**,**F**) show the reduction in fluorescence intensity (HO-1 expression) in cultures treated with crocin for 24 h before exposure to NaN_3_ (**C**) and blue light (**F**). Nuclei were counterstained with DAPI. (Scale bars: 200 µm).

**Figure 2:**
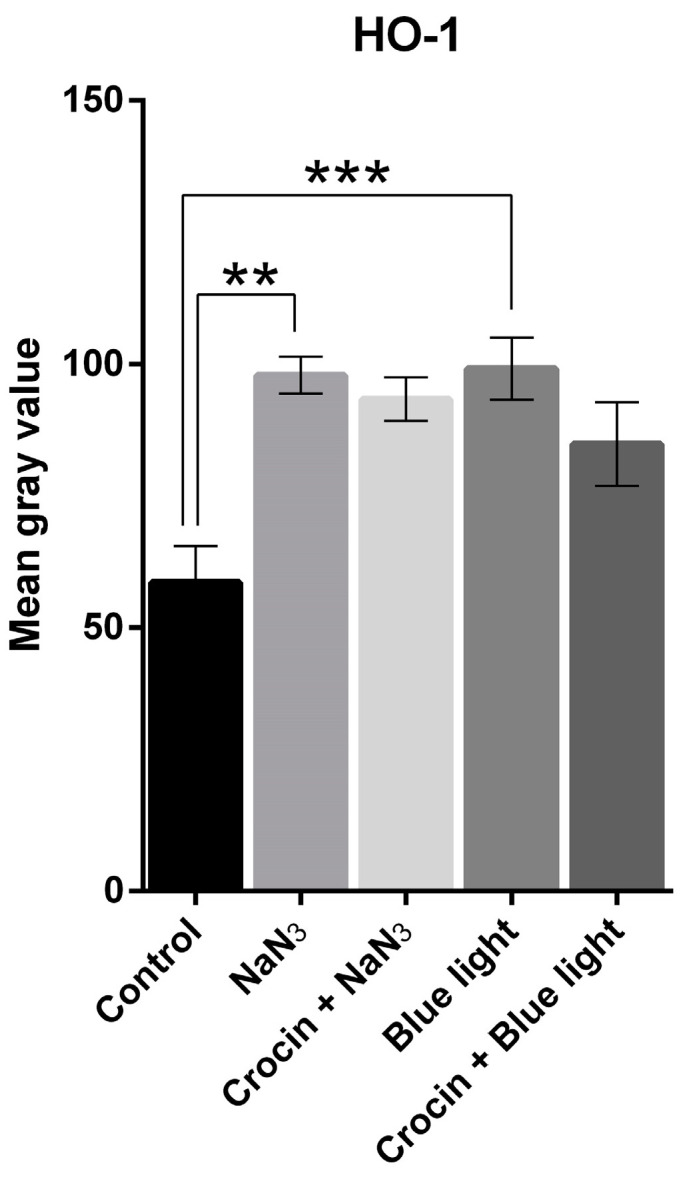
Graph shows the distribution of mean gray values (scaled 0 to 256) corresponding to the expression of HO-1 upon stimulation with NaN_3_ and blue light compared with crocin-pretreated cultures. A one-way ANOVA with Tukey’s multiple comparisons test was used for parametric data. (Statistical significance is represented by asterisks ** = *p* < 0.01; *** = *p* < 0.001).

We used densitometry analysis of HO-1 enzyme expression on immunofluorescence images to evaluate the occurrence and intensity of oxidative stress in trigeminal neuron primary cultures. Figure 1 shows that neurons in normal culture conditions exhibit a basal low intensity of HO-1 immunofluorescence signal (Figure 1A,D). Cultures exposed to NaN_3_ showed a strong increase in HO-1 expression as suggested by the significant elevation of the fluorescence signal intensity (*p* < 0.01; Figure 1E and Figure 2). This increase was also observed when cells were exposed to a 470 nm light source (*p* < 0.001; Figure 1B and Figure 2), indicating that overexposure to blue light activates enzymatic systems related to antioxidant response.

Crocin-pretreated cultures also showed an increase in HO-1 expression compared to the control, similar to that registered in cultures exposed to NaN_3_ and blue light (Figure 1C,F and Figure 2), but was not statistically different from the control.

**Figure 3:**
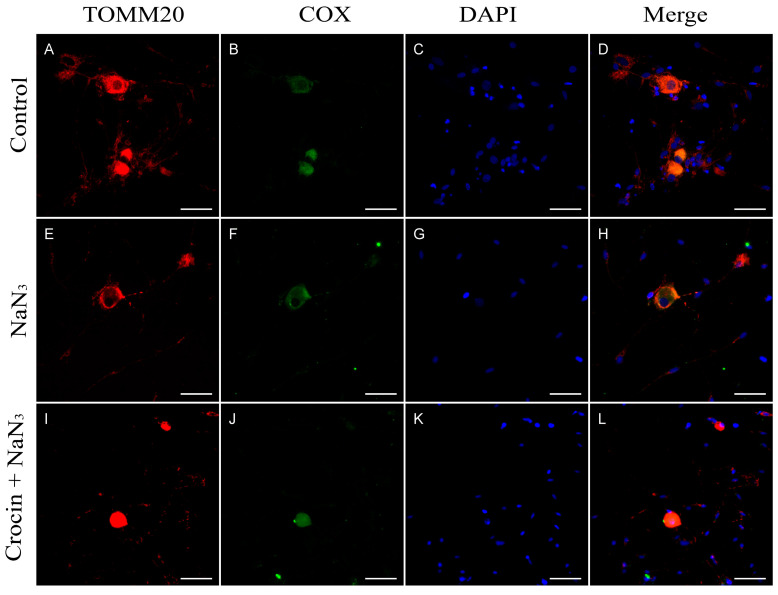
Immunostaining for TOMM20 and COX. (**A**–**D**) Neurons from primary cultures of trigeminal ganglia under normal conditions. (**E**–**H**) Cultures treated with NaN_3_ for 24 h. Note that in the case of cultures treated with NaN_3_, there is delocalization of cytochrome C and the mitochondrial marker TOMM20 (H). (**I**–**L**) Cultures pretreated with crocin before NaN_3_ damage. Nuclei were counterstained with DAPI. (Scale bars: 200 µm).

In addition, the effect of NaN_3_ on the mitochondrial structure was evaluated by the immunocytochemical study for the colocalization of cytochrome c oxidase enzyme (COX) with the mitochondrial membrane specific marker TOMM20 (Translocate of the Outer Mitochondrial Membrane 20). COX is present as a transmembrane protein of the mitochondria, thus coinciding with TOMM20 as seen by immunofluorescence (Figure 3D). After application of NaN_3_ to the cultures, the enzyme COX was supposed to be cleaved from the mitochondrial membrane and it was observed to be delocalized in the cell cytoplasm, often in peripheral regions (Figure 3H). The pretreatment with crocin (Figure 3L) prevented the delocalization of COX from the mitochondrial membrane.

**Figure 4:**
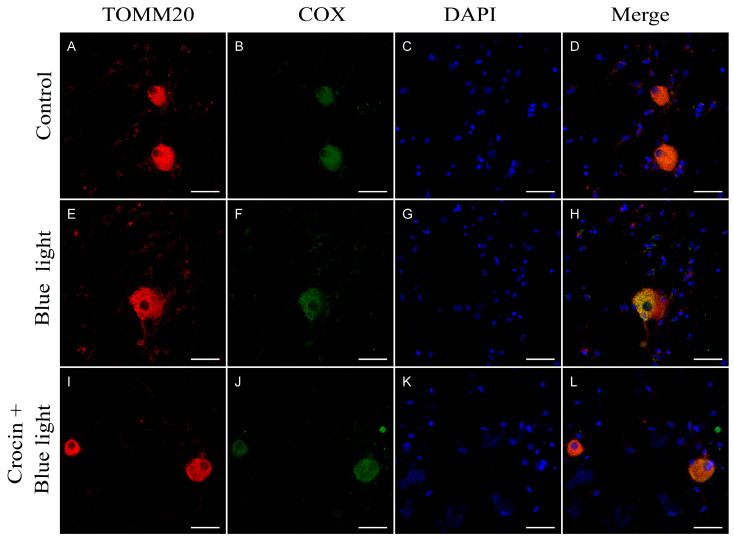
Immunostaining for TOMM20 and COX. (**A**–**D**) Neurons from primary cultures of trigeminal ganglia under normal conditions. (**E**–**H**) Cultures treated with blue light for 24 h. Note the cleavage of COX in cultures exposed to blue light (**H**). (**I**–**L**) Cultures pretreated with crocin before blue light exposure. Nuclei were counterstained with DAPI. (Scale bars: 200 µm).

Cultures exposed to blue light for 24 h also showed COX immunofluorescence out of the mitochondria (Figure 4H). Treatment with crocin protected from COX cleavage, showing a correct colocalization of COX and TOMM20 (Figure 4L).

### 2.2. Crocin Preserves Cytoskeletal Structure of Sensory Neurites in Culture under Oxidative Stimulus

**Figure 5:**
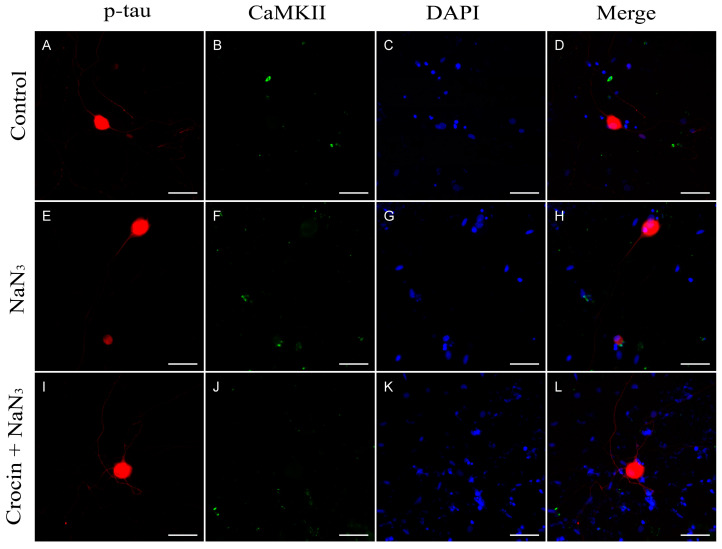
Expression of p-tau protein and CaMKII in neurons from primary cultures of trigeminal ganglia. (**A**–**D**) Images from control undamaged cultures showing normal morphology and level of expression of p-tau (**A**) and CaMKII (**B**). (**E**–**H**) Neurons exposed to NaN_3_ oxidative damage. Note the reduction in neurite number (**E**) and a slight increase in CaMKII immunofluorescence (**F**). Cultures pretreated with crocin (**I**–**L**) showed a significant higher number of neurites (**I**) and a basal expression of CaMKII (**J**). Nuclei were counterstained with DAPI. (Scale bars: 200 µm).

**Figure 6:**
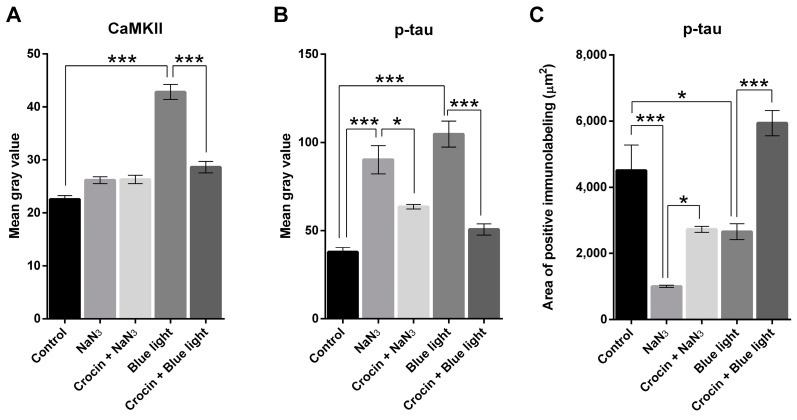
Graph (**A**) shows the distribution of mean gray values (scaled 0 to 256) corresponding to the expression of CaMKII upon stimulation with NaN_3_ and blue light compared with crocin-pretreated cultures. Graph (**B**) shows the distribution of mean gray values corresponding to the expression of p-tau protein upon stimulation with NaN_3_ and blue light compared with crocin-pretreated cultures. Graph (**C**) shows the relative density (area of positive elements/total area of the selection) corresponding to the expression of p-tau upon stimulation with NaN_3_ and blue light compared with crocin-pretreated cultures. A one-way ANOVA with Tukey’s multiple comparisons test was used for parametric data. (Statistical significance is represented by asterisks * = *p* < 0.05; *** = *p* < 0.001).

Under normal culture conditions, trigeminal neurons showed an extensive and regular arborization of neurites, without significant alterations in their length or thickness, i.e., neurites showed uniform smooth appearance, without swellings or interruptions (Figure 5A). We used the activation of the expression of the Calcium calmodulin-dependent protein kinase II (CaMKII) as a marker of the early onset of neurotubule disassembly. The addition of NaN_3_ to cultures induced a weak increase in the intensity of CaMKII immunofluorescence (Figure 5F), not significantly different from control basal values (Figure 6A).

As shown by p-tau immunofluorescence staining, cultures submitted to NaN_3_ showed a significant reduction in the extension of the neurite arborization (*p* < 0.001; calculated as the fraction of area of p-tau signal respecting the area of the image), while the mean gray value of the fluorescence indicator (as an indicator of protein expression) was increased (*p* < 0.001), suggesting an accumulation of p-tau protein at the soma or an increase in phosphorylated tau protein (Figure 5E and Figure 6B). In crocin-pretreated cultures (Figure 5I), the extension of the neurite loss was significantly lower than in NaN_3_ cultures (*p* < 0.05; Figure 6C). In addition, the amount of phosphorylated tau was lower in crocin-treated cultures compared with untreated (*p* < 0.05; Figure 6B).

**Figure 7:**
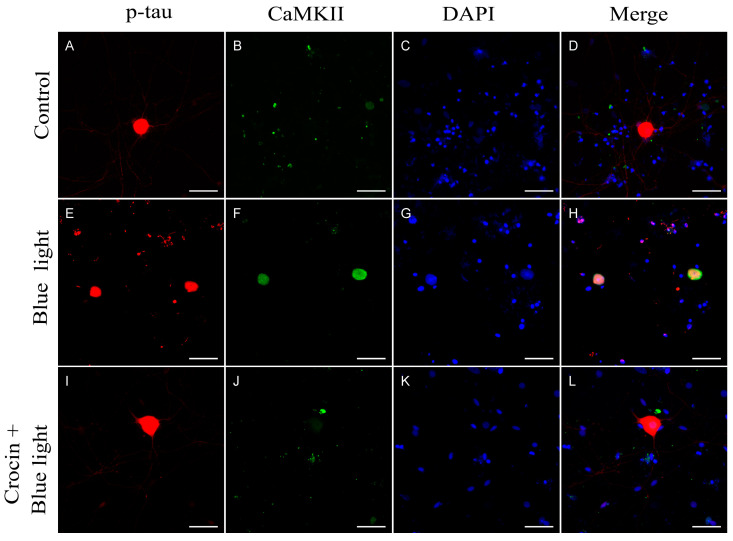
Expression of p-tau protein and CaMKII in neurons from primary cultures of trigeminal ganglia. (**A**–**D**) Images from control undamaged cultures showing normal morphology and level of expression of p-tau (**A**) and CaMKII (**B**). (**E**–**H**) Neurons exposed to blue light damage. Note the reduction in neurite number (**E**) and a sharp increase in CaMKII immunofluorescence (**F**). Cultures pretreated with crocin (**I**–**L**) showed a significant higher number of neurites (**I**) and a reduced expression of CaMKII (**J**). Nuclei were counterstained with DAPI. (Scale bars: 200 µm).

Blue light-exposed neurons in culture showed a sharp increase in CaMKII expression compared to the control cultures (*p* < 0.001; Figure 6A and Figure 7F) and NaN_3_ cultures (*p* < 0.001; Figure 6A and Figure 5F). This increase in CaMKII expression was not observed in cultures in which crocin were previously added (Figure 7J). We observed an intense increase in the mean gray value of p-tau immunofluorescence signal after blue light insult (*p* < 0.001), compared with the control cultures, accompanied by a significant reduction in neurite area (*p* < 0.05; Figure 7E and Figure 6B,C). Crocin-treated cultures showed a reduction in p-tau expression compared with blue light-exposed neurons (*p* < 0.001) that were together with the preservation of virtually the total density of neurite arborization of a control culture, compared with blue light-damaged neurons (*p* < 0.001; Figure 7I and Figure 6B,C).

**Figure 8:**
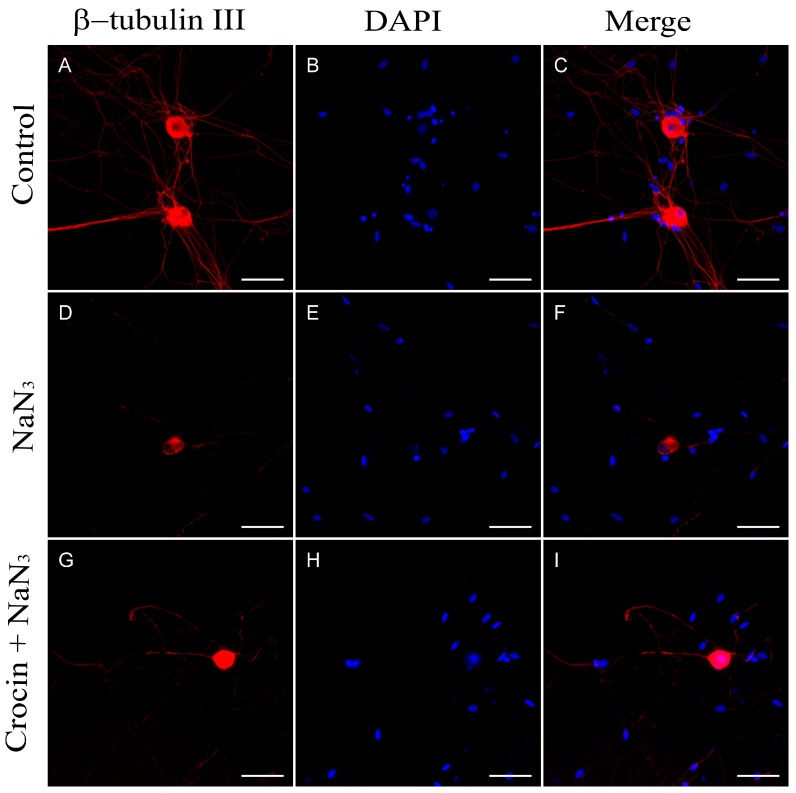
(**A**–**C**) Immunodetection of β-tubulin III in neurons from primary cultures of trigeminal ganglia under normal culture conditions. The addition of NaN_3_ to the culture medium induced abnormalities and a reduction in neurite density (**D**–**F**). Pretreatment with crocin for 24 h exerted a partial protective effect on the density of neuronal projections (**G**–**I**). Nuclei were counterstained with DAPI. (Scale bars: 200 µm).

We used a rabbit monoclonal antibody to neuronal class III β-tubulin (β-tubulin III) as a specific marker of neuronal microtubules in order to observe the alterations in the morphology of the neurites. NaN_3_ induces a drastic decrease in neurite number estimated as a significant reduction in the relative area immunolabeled by β-tubulin III (*p* < 0.001). In addition, axonal lesions were observed in the remaining neurites, usually showing swellings and axon breakdowns (Figure 8D).

**Figure 9:**
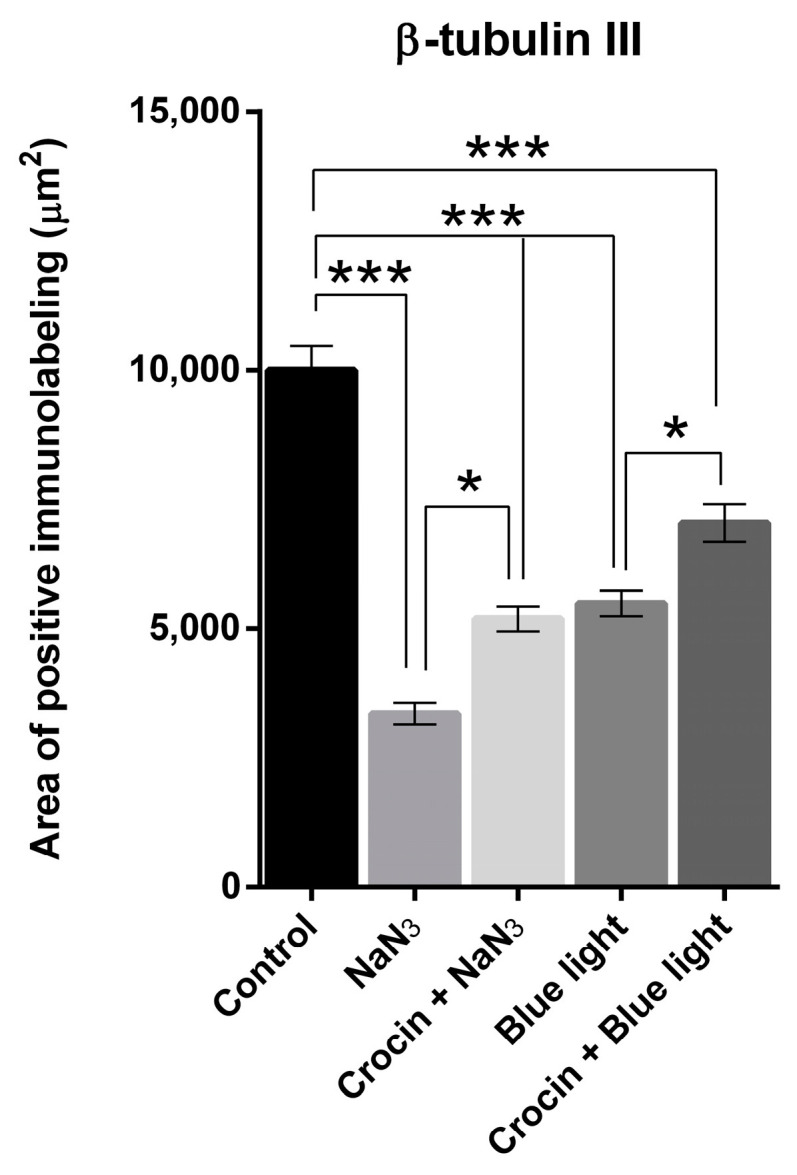
Graph shows the relative density (area of positive elements/total area of the selection) corresponding to the expression of β-tubulin III upon stimulation with NaN_3_ and blue light compared with crocin-pretreated cultures. A one-way ANOVA with Tukey’s multiple comparisons test was used for parametric data. (Statistical significance is represented by asterisks * = *p* < 0.05; *** = *p* < 0.001).

The addition of crocin to cultures previous to the NaN_3_ insult reduced the extension of neurite loss compared with those without antioxidant supplementation (*p* < 0.05; Figure 9). Regarding the morphology of neurites, engrossed fibers were visible throughout the culture as can be viewed in Figure 8G. However, very few neuron processes were identified as interrupted.

**Figure 10:**
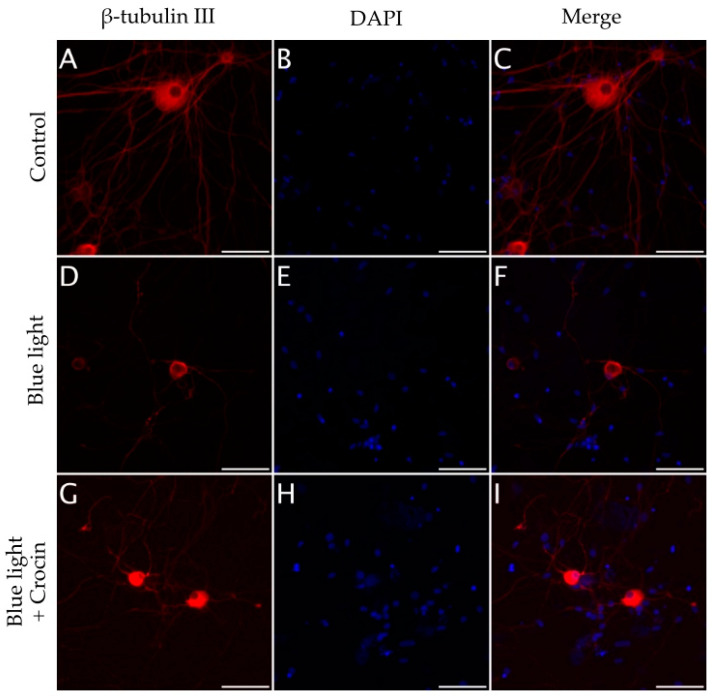
(**A**–**C**) Immunodetection of β-tubulin III in neurons from primary cultures of trigeminal ganglia under normal culture conditions. The exposure to blue light induced a reduction in neurite density (**D**–**F**). Pretreatment with crocin for 24 h exerted a partial protective effect on the density of neuronal projections (**G**–**I**). Nuclei were counterstained with DAPI. (Scale bars: 200 µm).

Blue light overexposure induced a significant decrease in the density of neurites (*p* < 0.001) compared with cultures maintained under normal conditions (see Figure 9 and Figure 10A,D). The effect of crocin in the preservation of the neuronal structure was evident in view of the extension of β-tubulin III positive area. The area of β-tubulin III labeling in crocin-treated cultures was significantly higher to that of blue light-exposed cultures (*p* < 0.05), although it was also significantly lower than in the control cultures (*p* < 0.001; Figure 9).

### 2.3. Crocin Protects Corneal Innervation from Blue Light Overexposure In Vivo

**Figure 11:**
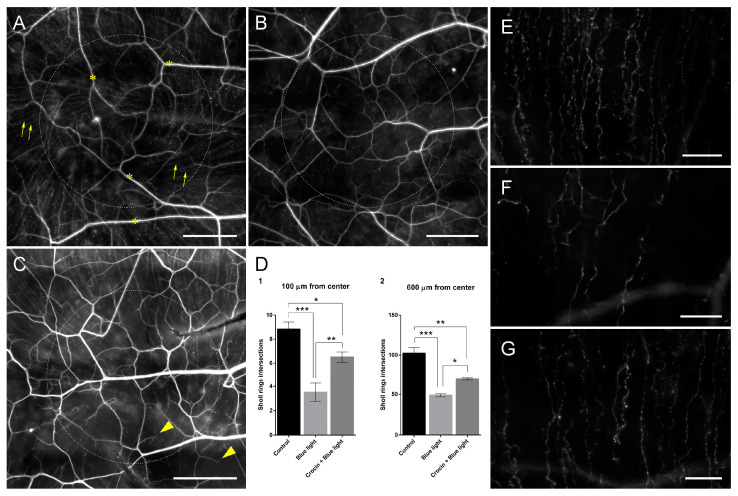
Whole mount preparations of rat corneas showing the corneal innervation of a control undamaged animal (**A**), a cornea exposed to blue light for 4 h over 10 days (**B**) and a cornea pretreated with topical 0.1 mM crocin over 3 days previous to blue light exposure (**C**). Arrows in (**A**) indicate sub-basal nerve fibers. Asterisks in (**A**) indicate stromal nerve bundles. Note the significantly higher density of sub-basal nerve fibers in (**C**) compared with (**B**) (see also (**D**)). Arrowheads in (**C**) indicate regenerative milieu at the tip of sub-basal nerve fibers. β-tubulin III immunofluorescence signal was artificially colored to gray in order to increase the visualization of the small sub-basal fibers. Dotted circles indicate the 600 µm Sholl ring. (**D**) Graphs showing the frequency of intersections of sub-basal nerve fibers with Sholl rings at 100 and 600 µm from the center. A one-way ANOVA with Tukey’s multiple comparisons test was used for parametric data. (**E**) Detail of sub-basal nerve fibers from image (**A**) (control undamaged) at the level of the 600 µm Sholl ring. (**F**) Detail of sub-basal nerve fibers from image (**B**) (exposed to blue light) at the level of the 600 µm Sholl ring. (**G**) Detail of sub-basal nerve fibers from image (**C**) (pretreated with crocin before blue light exposure) at the level of the 600 µm Sholl ring. Note the difference in fiber density between groups. (Scale bars in (**A**–**C**): 200 µm; scale bars in (**E**–**G**): 50 µm; statistical significance is represented by asterisks in (**D**): * = *p* < 0.05; ** = *p* < 0.01; *** = *p* < 0.001).

**Figure 12:**
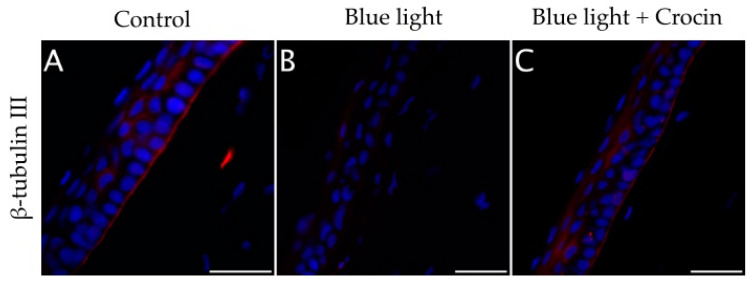
(**A**) Cross-sections of the cornea of an undamaged control rat showing a normal distribution of β-tubulin III positive sub-basal nerve plexus at the level of the basement membrane of the corneal epithelium. (**B**) Corneas exposed to blue light showed a loss of β-tubulin III immunolabeling at the basal epithelium, indicating nerve degeneration. (**C**) Corneas treated with crocin before blue light insult showed a visible β-tubulin III positive sub-basal plexus. Nuclei were counterstained with DAPI. (Scale bars: 50 µm).

In view of the results above, we performed an in vivo study to evaluate the potential damage of blue light overexposure on the corneal innervation of the rat. Also, we evaluated the neuroprotective capacity of a topical treatment with crocin. We used β-tubulin III immunofluorescence to visualize sub-basal nerve fibers on whole mount preparations of rat corneas, to analyze the variation in the density of nerve fibers in each group. Rats exposed to a source of 18 W/m^2^ blue light showed a significant reduction in the density of sub-basal nerve fibers compared with the control rats (Figure 11A,B and graphs in Figure 11D; *p* < 0.001). The loss of sub-basal fibers was evident at the central cornea where blue light induced the degeneration of the majority of the fibers, inducing the disorganization of the vortex and a drastic reduction in the number of individual sub-basal axons (Figure 11B,F). Corneas from rats treated topically with 0.1 mM for 3 days before the onset of blue light exposure also showed a decrease in the density of sub-basal nerve fibers compared with the controls. However, the amount of nerve fibers in crocin-treated corneas was significantly higher than in damaged corneas, both at 100 µm (*p* < 0.01) and 600 µm from the center of the cornea (Figure 12B,D,F).

The loss of elements of the sub-basal nerve plexus was also observed in cross sections of the cornea of blue light-exposed rats (Figure 12B) in comparison with a control rat (Figure 12A). Crocin-pretreated corneas showed positive β-tubulin III immunolabeling at the level of the sub-basal plexus, just beneath the epithelium, indicating a partial neuroprotective effect for 0.1 mM crocin applied topically (Figure 12C).

## 3. Discussion

Our results suggest that blue light plays a role in the induction of corneal sensory nerve degeneration under experimental conditions, demonstrated both in vitro and in vivo. Some evidence on the effect of blue light on the function and structure of corneal innervation were recently published by Gao et al. [22] using a mouse model, in which they described the generation of corneal pain, together with a decrease in β-tubulin III positive sub-basal nerve fibers in whole mount preparations. These results support our findings regarding the reduction in nerve density in the cornea of the rat after blue light overexposure.

There is an increasing interest in elucidating the effects of short-wavelength light on ocular health [33,34,35]. Our study agreed with recent works describing the effects of blue light on peripheral sensory nerves in the cornea [22,35] and is the first to propose a neuroprotective therapy specifically directed at corneal innervation using the antioxidant properties of crocin saffron derivative.

In order to elucidate the cellular mechanisms involved in corneal sensory nerve degeneration upon blue light exposure, we performed an in vitro model of rat trigeminal sensory neurons. We showed here that the damaging effect of blue light on trigeminal neurons was related to mitochondrial dysfunction caused by oxidative stress imbalance, as suggested by many others using other neuronal types [10,17,34]. 

HO-1 is a highly inducible stress-response protein that is expressed in situations of oxidative stress. HO-1 is an initial activator of oxidative stress, but also acts as a mechanism for regulating oxidative damage and maintaining cellular homeostasis [36]. The magnitude of HO-1 induction is now known to play a role in defending organisms against oxidative stress-mediated injuries and other diverse factors [37,38,39]. It is also well known that blue light causes an upregulation of HO-1 mRNA accompanied by an increase in the quantity of the protein detected by western blot analysis [17]. We found striking increases of HO-1 expression in cells exposed to blue light, indicating that blue light was inducing oxidative stress responses in the cells under our experimental conditions. The same activation of the HO-1 enzyme system was observed when the cultures were placed in contact with NaN_3_, a potent oxidative stress inductor. NaN_3_ is a specific inhibitor of COX [40,41], the mitochondrial complex IV of the electron transport chain, which is a key component in the function of the organelle, responsible for the oxidative phosphorylation to obtain ATP [42]. By means of colocalization immunofluorescence analysis, we observed that cells under NaN_3_ toxicity showed COX protein cleavage from the mitochondrial membrane (Figure 3H), suggesting alterations in the structure and function of mitochondria. Neurons exposed to blue light resembled the same delocalization of COX with mitochondrial marker TOMM20 (Figure 4H). The effect of blue light on mitochondria was described previously in retinal ganglion cells [10,17], and was also suggested in trigeminal ganglion neurons [18].

Impairment of mitochondrial functions lead to increased ROS levels as well as vulnerability to oxidative stress, and accumulation of ROS has long been associated with human neurodegenerative diseases [14]. Regardless of the trigger of the insult, which could be toxic, mechanical, metabolic or genetic, degeneration of axons and dendrites shares a common mechanism involving mitochondrial dysfunction and production of ROS [43,44]. Interdependent mitochondrial and cytoskeletal processes are central to axon survival, and impairment of these processes by injury or disease leads to pathological axon degeneration [45]. Reduced ATP production in the mitochondria is responsible for the failure of the overall functioning of the cell, starting with the destabilization of the cytoskeleton [46,47]. In this regard, a decrease in the amount of free ATP and GTP are directly related to microtubule instability [48,49] through the participation of the phosphorylation of the tau protein, involving kinases such as CaMKII [50]. The phosphorylated tau protein cleaves from the microtubule and the microtubule loses stability and begins to disassemble [47,51]. This molecular process occurs in axons and dendrites of degenerating sensory neurons [47,50].

In our cultures, sensory neurons damaged by either NaN_3_ or blue light showed significant elevations of p-tau expression, as interpreted from the variation in the mean gray values respecting basal intensity of the control cultures (Figure 5E, Figure 6B and Figure 7E). This increase in p-tau was accompanied by a sharp increase in CaMKII expression in the case of blue light insult, while a lower increment was recorded in NaN_3_-damaged cells. This discrepancy may be explained by the target of each insult. While NaN_3_ is affecting primarily to complex IV of the electron transport chain [40,41], blue light may be absorbed by other flavonoids and cytochromes in the chain, for instance, complexes I and II [10], elevating the potential impact on mitochondrial function caused by extraordinary doses of this light spectrum. In addition, endoplasmic reticulum may be involved in blue light phototoxicity [18].

In accordance with the dysfunctional mitochondrial-driven axonal degeneration, our results showed that neurons submitted to oxidative insults (NaN_3_ and blue light) exhibited degenerative signs in their neurites, as indicated by a decrease in neurite density and the presence of fiber abnormalities. These observations were made through the study of p-tau and β-tubulin III immunolabeling in vitro, but were also reproduced in vivo in rats overexposed to blue light for 10 days. We observed a severe reduction in corneal nerve density compared with the undamaged control rats in a short period of 10 days. The loss of sub-basal axons has been previously reported using a similar source of blue light on mouse models [22,35]. In contrast to Gao’s work, we used an LED source emitting at 470 nm instead of 420 nm. In addition, our dosage of exposure was set to 18 W/m^2^, 4 h per day for 10 days, while Gao’s mice were exposed to 2.722 mW/cm^2^ cycles of 12 h alternating light exposure and darkness for 14 days. Marek et al. used devices with 6 mW/m^2^ and analyzed corneal innervation using in vivo corneal confocal microscopy [35]. Despite the methodological differences, it is noteworthy that blue light seems to have an effect on the disturbance of the homeostasis of corneal innervation under experimental conditions, and that an overdose of high intensity SWL may also have an effect on the ocular surface of patients experiencing diseases related to oxidative stress or aging.

Axonal degeneration represents an early pathological event in neurodegeneration, constituting an important target for neuroprotection [43]. Corneal innervation impairment is usually related to loss of tear film regulation [5,6], neuropathic pain, epithelial lesions and corneal ulceration [52,53]. Treatments to restore ocular surface homeostasis include the use of artificial tears, with good outcomes regarding lubrication and relief of desiccation symptoms [54,55,56]. However, they are ineffective in the protection or recovery of the impaired corneal innervation [57,58]. Alternative topical methods, including autologous plasma rich in growth factors (PRGF^®^; BTI, Vitoria, Spain) [59,60,61], and synthetic heparan sulfate mimetic polymers (RGTA Cacicol^®^; Théa Laboratoires, Clermont-Ferrand, France) [62,63] have been shown to improve ocular surface wound healing and nerve regeneration in animal models. Antioxidants have been assayed in the ocular surface as anti-inflammatory therapy [24] and for the treatment of DED [64] but, to date, no treatment has focused specifically on the neuroprotection of sensory corneal innervation.

Our work demonstrates a potent neuroprotective effect of the saffron derivative crocin on the sensory projections of the trigeminal ganglion neurons innervating the cornea. In our experiments, crocin was effective in maintaining the expression of HO-1 in vitro under damaging conditions. As suggested by other authors, crocin may induce an upregulation of HO-1 aimed to activate the antioxidant responses against oxidative stress, suppressing the activity of nitric oxide and, for example, protecting from methotrexate-induced renal oxidative damage in rats [32,65]. 

Two of the main mechanisms of crocin in scavenging free radicals have been proposed: (1) hydrogen atom transfer (HAT) and (2) single electron transfer (SET). In the HAT mechanism, crocin deactivates free radicals by transferring a hydrogen atom to the free radicals. In another mechanism, SET, crocin neutralizes free radicals by donating an electron to the free radicals [32,66]. We have observed that crocin protects mitochondrial function in a highly efficient manner, preventing, in a significant part, the cleavage of the enzyme COX from the mitochondrial membrane (Figure 3L and Figure 4L). This feature may indicate the preservation of the function of the mitochondria and would contribute to a better management of ROS generated by blue light and NaN_3_. The reduction in CaMKII expression to basal levels in crocin-pretreated cultures may also contribute to maintaining the cytoskeleton stabilization by the action of the tau protein at the neurotubules. Our observations, in this sense, demonstrate a higher density of neurites in vitro in crocin-treated cultures compared with neurons exposed to damage.

Several works have reported the effect of crocin and saffron derivatives with the central nervous system in different neurodegenerative diseases, such as Alzheimer’s or Parkinson’s diseases, where it effectively scavenges free radicals, decreases the development of peroxidized membrane lipids and restores regular SOD activity [25,32]. These facts support our results, suggesting a neuroprotective role of crocin against different forms of oxidative damage in corneal innervation. 

Among saffron derivatives, picrocrocin and safranal are relatively unstable as they are transformed by dehydration under normal storage conditions and lose their concentration-dependent properties [67]. Safranal has been reported to be cytotoxic at low concentrations [68]. Crocetin is probably the molecule with the most potent active effect in the organism [67,69]. However, it has a low solubility in aqueous media, which affects its biodistribution. On the other hand, crocin is a carotenoid with a high solubility in water (10 µg/mL; 10 times more than crocetin). Crocin 1 (α-crocin) is a diester of crocetin with 2 gentiobiosyl esters and represents 70% of the crocins in saffron, and it is easily hydrolyzed to crocetin [67]. It has been described by Song and others [69,70,71] that crocetin may be the active component potentially responsible for the pharmacological effect of crocin, since orally administered crocin is rapidly converted to crocetin in the gastrointestinal tract, and the plasma concentration of corcetin was found to be higher than that of crocetin itself after equal molar oral administration of crocin [71,72]. Water-based ocular administration of crocin is preferable to that of crocetin due to the poor dissolution of the crocetin in aqueous fluids. 

In this work, the neuroprotective role of crocin was also observed in whole mount preparations of corneas from animals that were exposed to blue light, with a significant improvement in the density of corneal nerve fibers compared to blue light-exposed rats. Crocin seems to stimulate the sprouting of regenerative axons in the cornea. Taking all these premises together, we decided that the best choice for our study was crocin 1. Due to the large number of studies supporting the effect of crocin 1 on oxidative stress [32], we decided to select crocin 1 instead of other crocins for our study. Numerous studies have highlighted the beneficial properties of crocin 1 in in vivo neurodegenerative studies and in vitro models, where crocin 1 appeared to be more effective than other crocins (for a review see [69]).

To our knowledge, this is the first report of the efficacy of a topical treatment based on the saffron derivative crocin, directed specifically to the neuroprotection of the sensory innervation of the cornea. Therefore, we hypothesized that crocin 1 could be a potential candidate for developing a topical neuroprotective therapy for the ocular surface. However, further studies are needed to evaluate the effect of crocin in comparison to other antioxidant molecules with potential ocular application.

## 4. Materials and Methods

### 4.1. Animals and Experimental Design

A total of 33 adult male Wistar rats (*Rattus norvegicus*), obtained from Charles River Laboratories (L’Arbresle Cedex, France) and weighing about 300 g, were used for this work.

Fifteen rats were used to obtain primary sensory neuron cultures from trigeminal ganglia for the in vitro experiments. Three rats were used for each study group:

1. Control: undamaged cultured neurons maintained in normal culture conditions during the experiment.
2. NaN_3_ damage: cultures exposed to 5 mM NaN_3_ (Merck KGaA, Darmstadt, Germany) for 24 h.
3. Blue light damage: cultures exposed to irradiation with 18 W/m^2^ light stimulation with a source of 470 nm LED light.
4. Crocin + NaN_3_: sensory neuron cultures pretreated with 0.05 mM crocin (Merck) for 24 h before NaN_3_ damage.
5. Crocin + Blue light: sensory neuron cultures pretreated with 0.05 mM crocin (CAS 42553-65-1; Merck) for 24 h before blue light damage.

Before inducing damage (using NaN_3_ and blue light), crocin was removed by washing the medium and replacing it with fresh medium.

Eighteen additional rats were used for the in vivo experiments, consistent in the evaluation of the damaging potential of blue light on the corneal innervation and the neuroprotective effect of topical treatment with crocin. Three groups were designed, including 6 rats per group:

1. Control: undamaged rats maintained in dark conditions during the extension of blue light exposure.
2. Blue light exposure: rats exposed for 4 h over 10 days to an LED source of blue light (470 nm; 18 W/m^2^).
3. Crocin + Blue light exposure: rats treated with topical drops of 10 µL of 0.1 mM crocin, for 3 days before blue light exposure.

Prior to inducing damage with NaN_3_ and blue light, the ocular surface was thoroughly washed three times with BSS (BSS, Sterile Irrigating Solution, Alcon Laboratories, Inc., Fort Worth, TX, USA) to remove any potential crocin residues. See Supplementary Material S1 for a graphical summary of the in vitro and in vivo experiments. See Supplementary Material S2 for the dose-response curves used to establish the concentrations of NaN_3_ and crocin for these experiments. 

The animals were kept, handled and sacrificed according to the Statement for the Use of Animals in Ophthalmic and Vision Research of ARVO and the guidelines established in the Directive of the European Parliament and of the Council of the European Communities (2010/63/EU), of the Spanish legislation (RD 53/2013), in force for the use and care of laboratory animals, and the procedures were carried out under the approval of the Ethical Committee of the University of Oviedo (PROAE 27/2021 of 20 October 2021).

### 4.2. Blue Light Devices

The blue light-emitting devices were designed and made in the laboratory. Blue LED bulbs of 5 mm (12.675/5/AZ/C/SL, Electro DH S.L., Hospitalet de Llobregat, Spain) were assembled with each other and with resistors of 56 amperes, following a given circuit. They were connected to power by using 12 V power supplies.

### 4.3. Primary Cultures of Trigeminal Sensory Neurons

On the day of sacrifice, animals were euthanized by sodium pentobarbital overdose (Dolethal, Vétoquinol, Lure, France) injected intraperitoneally, under general anesthesia. The trigeminal ganglia were excised from euthanized rats and processed for cell culture. The ganglia were enzymatically digested with 0.125% collagenase type II (Gibco, Grand Island, NY, USA) in Leibovitz L15 culture medium (Gibco) at 37 °C for two hours. Cells were resuspended in a medium containing Neurobasal-A (Gibco); 2% Supplement B27 (Gibco); 50 ng/mL NGF 2.5S (Merck); 2 mM L-Glutamine (Gibco) and 1% antibiotics (penicillin, streptomycin and azithromycin; Merck). Primary cultures of trigeminal ganglia contained a heterogeneous mixture of cell populations, including sensory neurons, satellite and Schwann cells, and sporadic fibroblasts from the connective capsule. To avoid proliferation of non-neuronal cells in the culture, 7.5 µg/mL fluorodeoxy-uridine and 17.5 µg/mL uridine (both from Merck) were added to the medium. Cells were plated in 96-well culture plates (Nuncon Delta Surface; Thermo Fisher Scientific, Boston, MA, USA) previously coated with 100 µg/mL poly-l-lysine (Merck) and 100 µg/mL Laminin (Merck). Medium was changed every three days.

The cultures were maintained for 6 days until the neurons exhibited a complete dendritic arbor.

### 4.4. Fluorescence Immunocytochemistry 

#### 4.4.1. Immunofluorescence on Cell Cultures

At the end of the experiments, cultures were fixed in 4% paraformaldehyde for 10 min. The following antibodies were used at the dilutions: mouse monoclonal anti-cytochrome c oxidase (1:500; Abcam, Cambridge, UK); rabbit polyclonal anti-TOMM20 (1:1000; Abcam); rabbit monoclonal anti-p-tau (1:100; Abcam); rabbit monoclonal anti-beta3-tubulin (1:200; Cell Signaling Technology, Danvers, MA, USA); mouse monoclonal anti-CaMKII-alpha (1:500, Cell Signaling Technology); rabbit polyclonal anti-Hemoxigenase 1 (1:100, Enzo Biochem, Farmingdale, NY, USA). For anti-cytochrome c oxidase immunofluorescence assay, antigen unmasking was performed using urea buffer (5% urea in 0.1 M Tris-HCl, pH 9.5) for 10 min. Unmasking of anti-CaMKII was also performed with 100% methanol for 10 min. Antibodies were incubated with the cells overnight at 4 °C in a medium containing 0.05% Tween 20, 5% normal goat or donkey serum and PBS 0.1 M pH 7.4. The immunodetection was revealed using 488 and 594 AlexaFluor rabbit and goat complementary secondary antibodies against mouse and rabbit IgG, respectively (Molecular Probes, Eugene, OR, USA), incubated for 2 h at room temperature (RT) and protected from light. Finally, nuclear counterstaining was performed using 2 µg/mL DAPI (4′,6-diamidino-2-phenylindole; Molecular Probes).

#### 4.4.2. Immunofluorescence on Tissue: Whole Mount Corneal Preparation

Freshly enucleated rat eye globes were immediately fixed in 4% paraformaldehyde for one hour at RT. They were then dissected, and the cornea was carved. The cornea was refixed in 4% paraformaldehyde for one additional hour at RT. After that, the corneas were washed three times for 10 min with PBS and blocked for 1 h with 5% BSA, 5% goat serum, 0.2% sodium azide and 0.3% Triton X-100 in PBS (PBS-Triton; all products from Merck). Corneas were incubated for 24 h at RT in the presence of rabbit anti-neuronal class III tubulin (1:500; Abcam). The samples were then incubated for 24 h at RT in the presence of anti-rabbit IgG Alexa Fluor 594 secondary antibody (1:500; Molecular Probes). Afterwards, samples were rinsed three times with washing solution followed by incubation for 10 min at RT with DAPI, and, finally, they were mounted with coverslips and fluorescent mounting medium (DAKO, Glostrup, Denmark).

### 4.5. Microscopy and Image Analysis

Culture plates and whole mount preparations were observed and documented using a Leica DMI6000 inverted fluorescence microscope and a Leica DM6000B fluorescence microscope, respectively, equipped with a digital image capture system (LASX, Leica Microsystems GmbH, Wetzlar, Germany) and a Leica DFC310 FX camera (Leica Microsystems).

Image analysis of neurons in culture was performed using FIJI software (ImageJ 1.54f, National Institutes of Health, Bethesda, MD, USA). The optical density and the area of the expression of the different markers were analyzed automatically on thresholded images. Mean gray value automatic analysis was used to estimate the level of expression of a fluorescent probe in segmented cells in a 0 to 256 scale. The expression value for each antibody was obtained as the average of the results obtained from the analysis of each individual neuron. Area and % Area was used to estimate the surface covered by each marker. A minimum of 40 neurons per well from 3 wells of each one of the 3 replicas were measured for each experimental condition, representing 12.80% of the total number of neurons. See Supplementary Material S3 for a detailed explanation of image analysis.

For Sholl analysis, images from whole mount corneas were acquired at 20× magnification. Leica LAS X software was used to fuse the adjacent tiles and produce maximum intensity projections. Adjacent image tiles were captured with overlap to ensure proper tiling using the Tile Scan tool. All images were acquired using the same intensity settings. ImageJ Sholl analysis v3.2.7 plugin from FIJI [73] was used to calculate the number of regenerative sensory axons in the injured region. Intersecting fibers were automatically counted at 100 and 600 µm from the corneal center.

### 4.6. Statistical Analysis

GraphPad Prism 8 statistical analysis software (GraphPad Software Inc., San Diego, CA, USA) was used for statistical analysis and for the generation of the graphs. Results obtained from image analysis of neuronal cultures and Sholl analysis was analyzed by a one-way ANOVA with Tukey’s multiple comparisons test. All data passed the Kolmogorov–Smirnov normality test. A *p* value of <0.05 was considered statistically significant. Data was expressed as mean ± SEM.

## Data Availability

The data presented in this study are contained within the article.

## Supplementary Materials

The following supporting information can be downloaded at: https://www.mdpi.com/article/10.3390/molecules29020456/s1, Supplementary Material S1: Experimental design: visual summary; Supplementary Material S2: Establishment of the concentration of use of sodium azide and crocin based on WST-1 cell viability assays (Cellpro-RO; Roche); Supplementary Material S3: Description of the image analysis.
