# Supplementary material for: Immunocytochemical Analysis of Crocin against Oxidative Stress in Trigeminal Sensory Neurons Innervating the Cornea
Source: Molecules. 2024 Jan 17;29(2):456. doi: 10.3390/molecules29020456 (PMC10818698; doi:10.3390/molecules29020456)
Supplement: Supplementary file 1 [file molecules-29-00456-s001.zip › molecules-2713480-supplementary.pdf]

S1.- Experimental design: visual summary

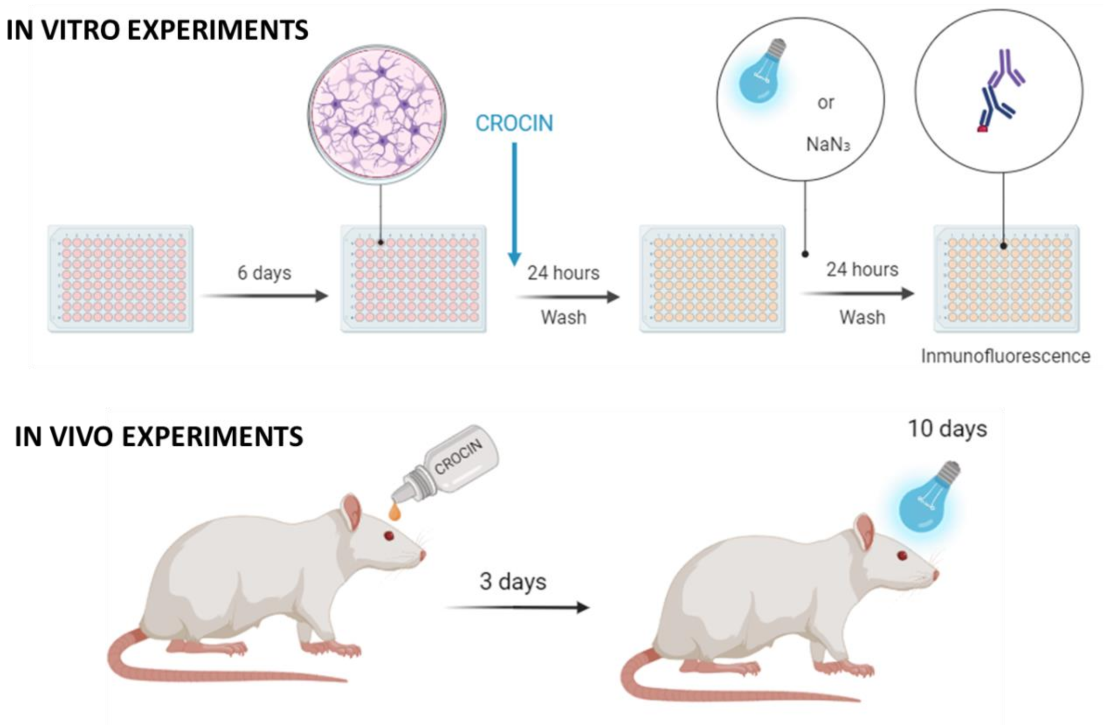

Figure S1: Summary of the sequence of procedures during *in vitro* and *in vivo* experiments.

S2.- Establishment of the concentration of use of sodium azide and crocin based on WST-1 cell viability assays (Cellpro-RO; Roche)

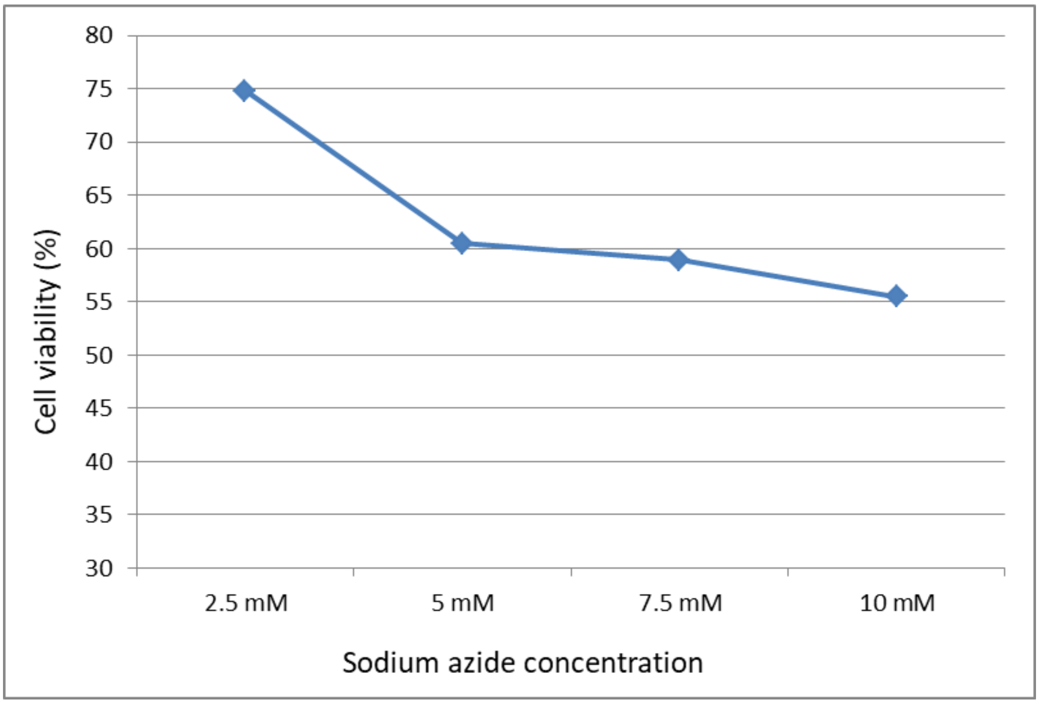

Figure S2: Viability curve of cultured neurons treated with different concentrations of sodium azide (WST-1 assay from Roche).

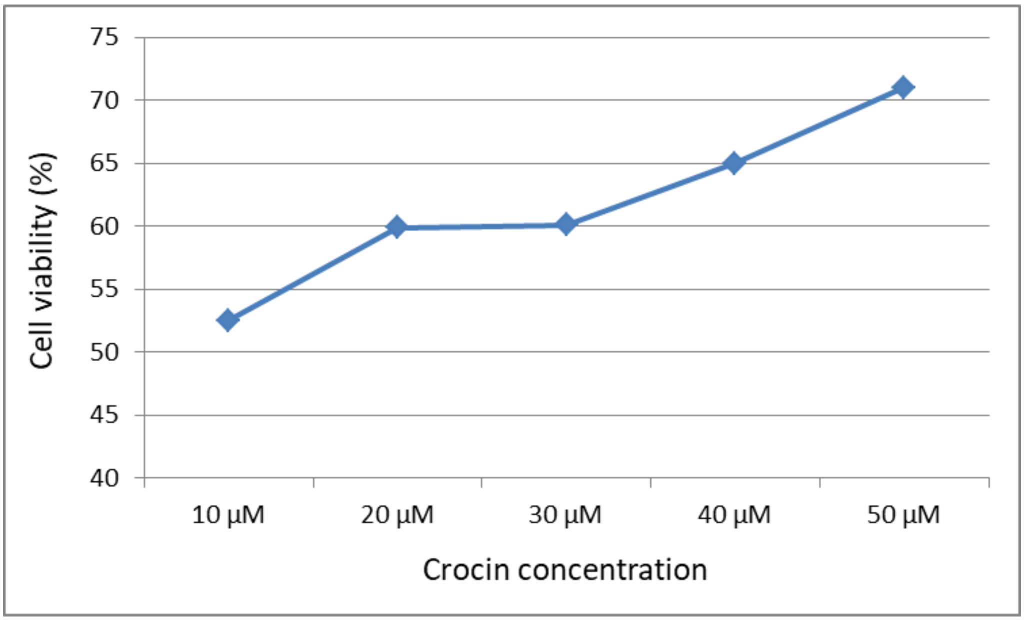

Figure S3: Viability curve of cultured neurons treated with different concentrations of crocin 1 (WST-1 assay from Roche).

S3.- Description of the image analysis

Using a Leica 6000I inverted fluorescence microscope and LASX software (both from Leica Microsystems), we captured images from primary cultures at 20X magnification (448.92 X 335.40 µm). Cells were homogeneously distributed throughout the culture well. We captured 30 images from each p96 well by randomly selecting areas using the random selection tool of the Tile Scan Leica LASX software, which was programmed to obtain non-overlapping images. Each image contained up to 5 neurons (interval 1 – 4; average 1.46 ± 0.13). This procedure allowed us to analyze at least 40 neurons from each well. We used 3 wells from each of the 3 identical plate replicas. A minimum of 360 neurons were measured in each experiment, representing 12.80% of the total number of neurons.

Each microscopy image was obtained by sequential acquisition of each channel. Each channel corresponded to a different fluorescent signal associated with each specific antibody or to DAPI-labeled nuclei. In this way, the LASX software captures an individual image for each channel and then merges them to obtain a composite image (Figure S4 B).

Images were subsequently analyzed using FIJI software (ImageJ 1.54f, National Institutes of Health, Bethesda, MD, USA). Optical density and the area labeled by different markers were automatically analyzed on calibrated and thresholded images. Each marker was analyzed separately using the “Split channels” command, resulting in a separate image for each marker, as shown in the figures of this article. Each of the images containing a measurable marker was automatically segmented using the “Threshold” command with fixed settings for each marker to detect only positive labeling and discard background. The images were then converted to binary images to obtain masks that define regions of interest (ROI) (Figure S4 C). The ROIs were then transferred to the colored image and we manually discarded all ROIs corresponding to non-neuronal cells (Figure S4 D).

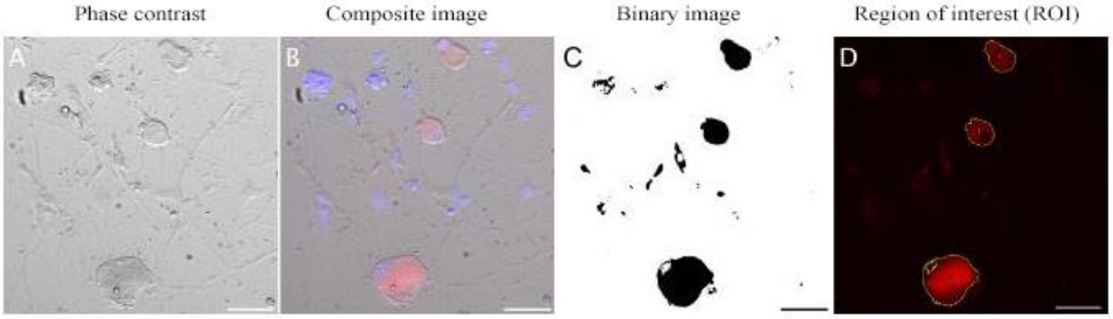

Figure S4: Example of image processing of a composite image using FIJI software. The example shows an image of a primary culture labeled with anti-HO-1 antibody. A binary mask is created from a thresholded image to select the regions of interest and transferred them to the colored image.

Once the ROIs of neurons were verified, automatic quantification of the “Mean Gray Value” and the percentage of labeled area (“% Area”) was performed by selecting these items in the “Set Measurements” command of the “Analyze” menu and by running the “Measure” command. The software generates an exportable table of results, including the mean gray value of all the pixels in the ROIs and the relative area labeled by the marker.

The mean gray value was used to estimate the level of expression of a fluorescent probe in segmented cells on a scale of 0 to 256. The expression value for each antibody was obtained as the average of the measurements of each pixel in each ROI, corresponding to a single neuron. Area and % Area were used to estimate the surface labeled by each marker. A minimum of 40 neurons per well from 3 wells of each one of the 3 replicas were measured for each experimental condition. We performed a background correction in our measurements by subtracting the mean value of an unlabeled ROI of the well from the mean value of the ROI measurements.

The results table was exported to an Excel file and transferred to a GraphPad Prism sheet for statistical analysis. Information from each image was considered as an individual value for statistical analysis using GraphPad Prism, with a minimum of 243 images per experimental condition.
